# Causes of Upper Gastrointestinal Bleeding Among Pilgrims During the Hajj Period in the Islamic Years 1437-1439 (2016-2018)

**DOI:** 10.7759/cureus.10873

**Published:** 2020-10-10

**Authors:** Bechayir M Youssouf, Bodor Alfalati, Reem Alqthmi, Rahma Alqthmi, Lina M Alsehly

**Affiliations:** 1 Internal Medicine, Umm Al-Qura University, Makkah, SAU

**Keywords:** upper gastrointestinal bleeding, hajj, pilgrims, causes, saudi arabia

## Abstract

Background

Approximately 3.5 million pilgrims perform Hajj every year. Planning for their healthcare requires knowledge of the pattern of diseases and risk factors of pilgrims who require hospitalization during the Hajj period. The aim of the current study was to evaluate common causes and risk factors of upper gastrointestinal bleeding (UGIB) among pilgrims during the Hajj season.

Method

We conducted a retrospective cross-sectional study using a data collection checklist to collect data from medical records. We included all patients who had UGIB and attended the endoscopy department of the King Abdulaziz Hospital, Mecca, in the AL-Hajj season of the Islamic years 1437-1439 (2016-2018).

Results

A total of 93 patients were included in the current study; of those, 65.59% were males. The mean age of the patients was 63.37 ± 12.83 years, and about one-third (29.03%) of them were Indonesian. Overall, melena with or without anemia was the most common presentation (44.09%), followed by hematemesis (34.78%), melena with hematemesis (15.05%), hematemesis with ascites (9.68%), and abdominal/epigastric pain (3.23%). The most common cause of UGIB was the presence of gastric origin (erosive gastritis/gastric ulcer/gastric tumor) with 22.58% of the patients presenting with the same. The most common factors were medications, especially non-steroidal anti-inflammatory drugs (NSAIDs) (37.63%) and blood thinners (22.58%). Hypertension (31.18%), diabetes mellitus (DM) (29.03%), and chronic liver disease/failure (27.96%) were the most common chronic conditions in the studied population.

Conclusion

Medical orientation towards high-risk pilgrims prone to developing UGIB who intend to travel may help reduce the risk of developing the condition, by taking proper measurements of those groups by the medical teams, especially in those with preventable factors.

## Introduction

Hajj, the yearly pilgrimage of Muslims to Mecca, is the world's largest peaceful mass gathering in terms of numbers, diversity of the population, and the regularity of different activities [1]. About 3.5 million pilgrims from about 200 countries travel to different holy places of Mecca, along with thousands of Saudi residents. This diverse population (in terms of ethnicity, race, gender, age, health, and socioeconomic status) performs the same activities over a limited period of time and a defined area of land, which is a challenge to the medical system due to high morbidity, less healthcare accessibility, and difficulty of evacuation in emergent cases [2].

Upper gastrointestinal bleeding (UGIB) is defined as bleeding from a source proximal to the Treitz ligament. UGIB is one of the most common causes of hospitalization in the world. During the Hajj pilgrimage, UGIB is one of the most common medical conditions seen among pilgrims as it has been estimated that 6.3% of all hospitalizations during the pilgrimage are due to UGIB [3].

Ulcer disease is the most common specifically identified cause of acute upper gastrointestinal hemorrhage. The peptic ulcer has been reported to be responsible for nearly 50% of cases of UGIB [4]. Consumption of aspirin and other non-steroidal anti-inflammatory drugs (NSAIDs) are among the most important predisposing factors of ulcer bleeding. The risk of gastrointestinal bleeding caused by NSAIDs appears to be dose-related. Another common risk factor is *Helicobacter pylori* *(H. pylori),* which is firmly linked to peptic ulceration [5].

Treating and preventing UGIB costs billions of dollars per year. The annual incidence of hospitalization for UGIB is one in 1,000 people in the United States, which is more frequent in men compared to women [4,6]. Mortality has fallen sharply in the last 30 years with the introduction of endoscopic therapy that reduces the rate of re-bleeding [7]. Moreover, increase in UGIB incidence has been attributed to the rising rate of its occurrence in older people who have a much worse prognosis than other patients due to their common use of platelet anti-aggregation factors or anticoagulants, as well as frequent comorbidities, with approximately 45% of patients hospitalized for UGIB being over the age of 60 years [8].

The aim of the current study is to evaluate the common causes of UGIB among pilgrims during the Hajj season between the Islamic years 1437-1439 (2016-2018).

## Materials and methods

Study design

This was a retrospective cross-sectional study using a data collection checklist to collect data from medical records. We included all patients who had UGIB and attended the endoscopy department of the King Abdulaziz Hospital, Mecca, during the AL-Hajj season of the Islamic years 1437-1439 (2016-2018). There were no restrictions regarding, age, gender, or nationality regarding the inclusion of patients in the study. A standardized data collection form was used to collect information on demographics (age, gender, and nationality), disease presentation, admission, and final diagnosis based on endoscopy results. Patients’ medical records were used to extract all data.

Statistical analysis

Data entry and analyses were conducted using SPSS Statistics V.26 (IBM, Armonk, NY). Mean and standard deviations (SD) were used to represent continuous variables, while we used frequencies and percentages to represent categorical variables. The Skewness-Kurtosis tests were used for testing the normal distribution of continuous variables. Chi-square test (or Fisher’s exact test, as appropriate) was used for categorical data; while a one-way analysis of variance (ANOVA) was used for continuous variables normally distributed, the Kruskal-Wallis H test was used for continuous variables not normally distributed. A p-value of <0.05 was considered to be statistically significant.

Informed consent and ethical considerations

The study was approved by the Ethical Committee of King Abdulaziz Hospital in Makkah (ID: H-02-k-076-0619-129). No identifying information of any patient was published and all collected data were only used for statistical analysis. Before the commencement, the study protocol was cleared by the institutional review board and the ethics committee. A waiver of consent was granted since the study was retrospective in nature.

## Results

Baseline characteristics

A total of 93 patients were included in the current study; of those, 65.59% were males. The mean age of the included patients was 63.37 ± 12.83 years, and about one-third (29.03%) of them were Indonesian. The Saudi nationality (18.28%) was the second in order, followed by Egyptian (9.68%), Bangladeshi (5.38%), Malaysian (4.3%), Indian (3.23%), and Syrian (3.23%) nationalities, respectively (Table 1).

**Table 1:** Baseline characteristics of the included patients *Statistically significant SD: standard deviation

Variable		Year								P-value
		1437 (n=11)		1438 (n=36)		1439 (n=46)		Total (n=93)		
		N	%	N	%	N	%	N	%	
Age, mean ± SD		59.27 ± 16.50		63.47 ± 14.17		64.26 ± 10.73		63.37 ± 12.83		0.515
Gender	Female	5	45.45	12	33.33	15	32.61	32	34.41	0.712
	Male	6	54.55	24	66.67	31	67.39	61	65.59	
Nationality	Afghan	0	0.00	1	2.78	1	2.17	2	2.15	0.006*
	African	0	0.00	1	2.78	0	0.00	1	1.08	
	Algerian	0	0.00	1	2.78	0	0.00	1	1.08	
	Athubian	0	0.00	1	2.78	1	2.17	2	2.15	
	Pakistani	0	0.00	0	0.00	2	4.35	2	2.15	
	Bangladeshi	0	0.00	2	5.56	3	6.52	5	5.38	
	Egyptian	1	9.09	5	13.89	3	6.52	9	9.68	
	Burmese	0	0.00	2	5.56	0	0.00	2	2.15	
	Burkinese	0	0.00	1	2.78	1	2.17	2	2.15	
	Indian	2	18.18	0	0.00	1	2.17	3	3.23	
	Indonesian	0	0.00	12	33.33	15	32.61	27	29.03	
	Jordanian	0	0.00	0	0.00	2	4.35	2	2.15	
	Libyan	0	0.00	0	0.00	2	4.35	2	2.15	
	Mali	0	0.00	0	0.00	2	4.35	2	2.15	
	Malaysian	0	0.00	2	5.56	2	4.35	4	4.30	
	Moroccan	0	0.00	1	2.78	0	0.00	1	1.08	
	Nigerian	0	0.00	0	0.00	2	4.35	2	2.15	
	Palestinian	0	0.00	0	0.00	1	2.17	1	1.08	
	Saudi	8	72.73	5	13.89	4	8.70	17	18.28	
	Sudanese	0	0.00	1	2.78	0	0.00	1	1.08	
	Syrian	0	0.00	0	0.00	3	6.52	3	3.23	
	Thai	0	0.00	0	0.00	1	2.17	1	1.08	
	‎Yemeni	0	0.00	1	2.78	0	0.00	1	1.08	

In terms of years, 46 patients were admitted in the Islamic year of 1439 (2018), 36 patients in 1438 (2017), and 11 patients in 1437 (2016). There was no statistically significant difference among patients’ characteristics in different years in terms of age (p = 0.515) and gender (p = 0.712). However, there was a statistically significant difference in nationality distribution across the years (p = 0.006) (Table 1).

Clinical presentation on admission

**Figure 1:**
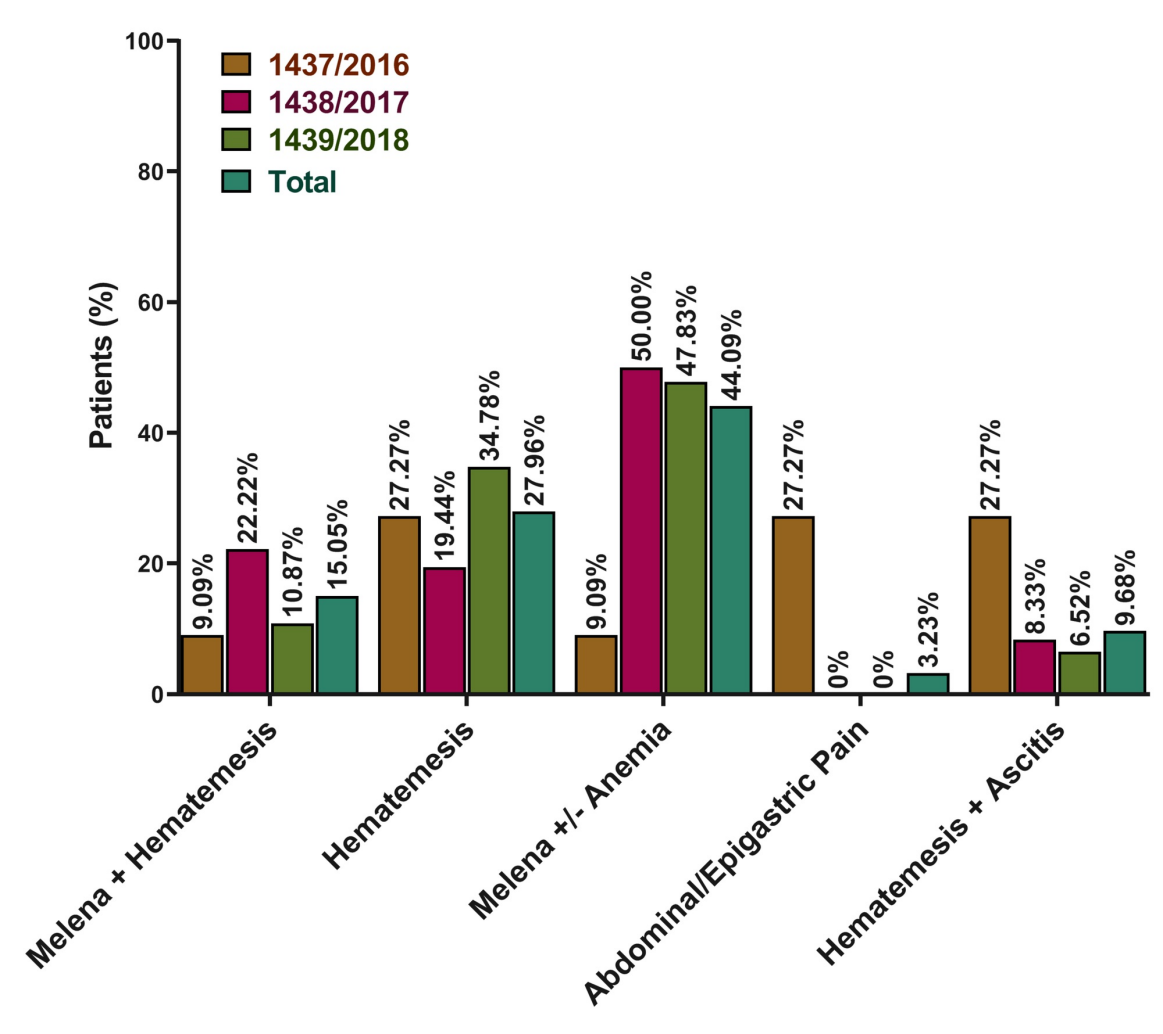
Distribution of different disease presentations among included patients (stratified by year)* *There was no statistically significant difference comparing different years (p-value = 0.052)

The majority (94.62%) of the patients were admitted through the emergency department, while only 5.38% of them were admitted through the outpatient clinic. Overall, melena with or without anemia was the most common presentation (44.09%), followed by hematemesis (34.78%), melena with hematemesis (15.05%), hematemesis with ascites (9.68%), and abdominal/epigastric pain (3.23%). In the Islamic year of 1437 (2016), hematemesis, melena with or without anemia, and abdominal/epigastric pain were all common presentations with an equal percentage of 27.27%. In the Islamic year of 1438 (2017), melena with or without anemia was the most common presentation (50%), followed by melena with hematemesis (22.22%), and hematemesis (19.44%). In the same context, melena with or without anemia was the most common presentation (47.83%) in 1439 (2018), followed by hematemesis (34.78%), and melena with hematemesis (10.87%), respectively (Figure 1).

Causes and risk factors

**Figure 2:**
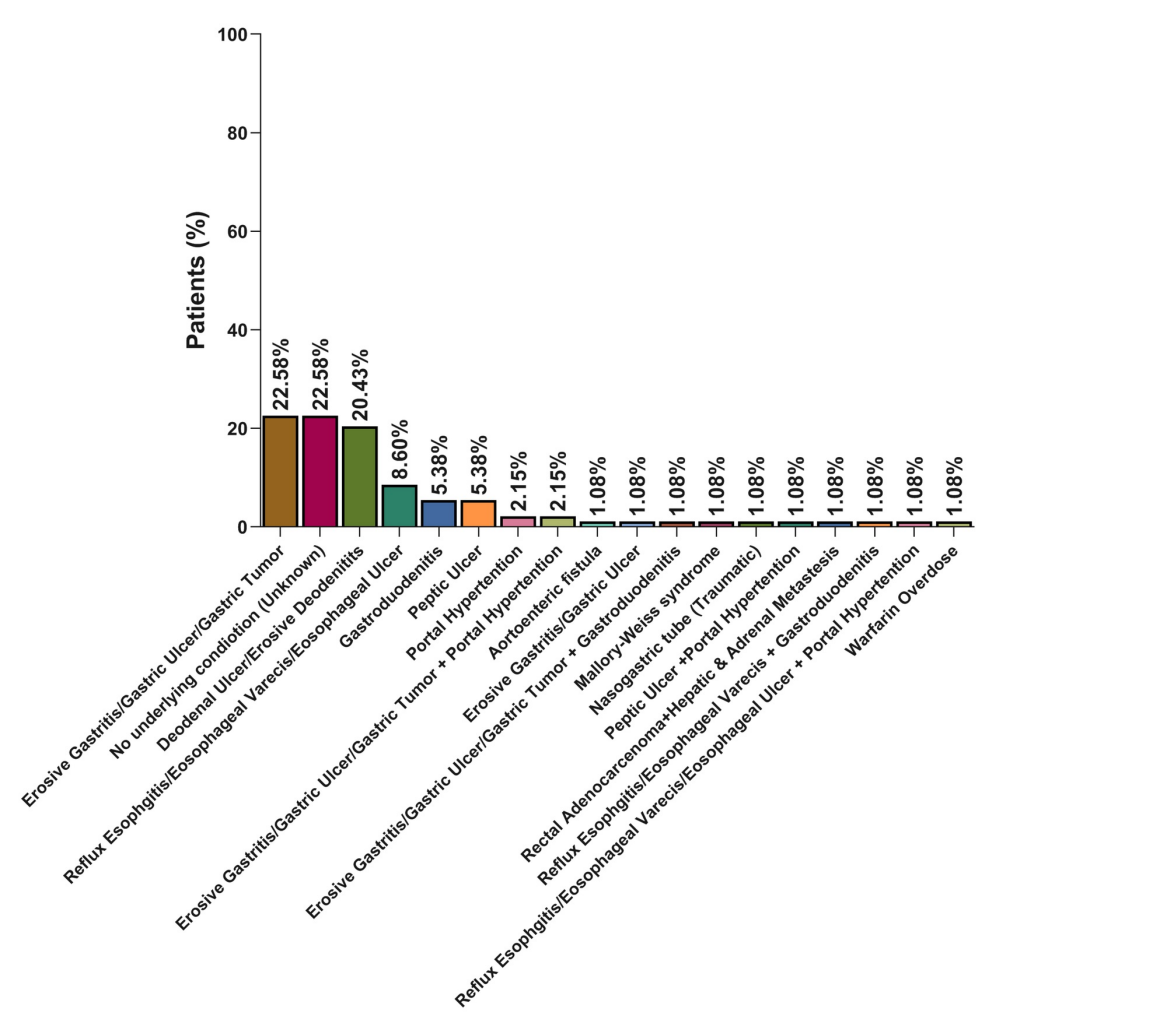
Distribution of different diagnoses (causes) among included patients

The most common cause of UGIB was conditions of gastric origin (erosive gastritis/gastric ulcer/gastric tumor) with 22.58% of the patients; however, the same percentage of the patients had UGIB of unknown origin/cause. The third most common origin of UGIB was duodenal (duodenal ulcer or erosive duodenitis) with 20.43% of the patients, followed by esophageal (8.60%), gastroduodenal (5.38%), peptic ulcer (5.38%), and portal hypertension (2.15%), respectively (Figure 2).

**Figure 3:**
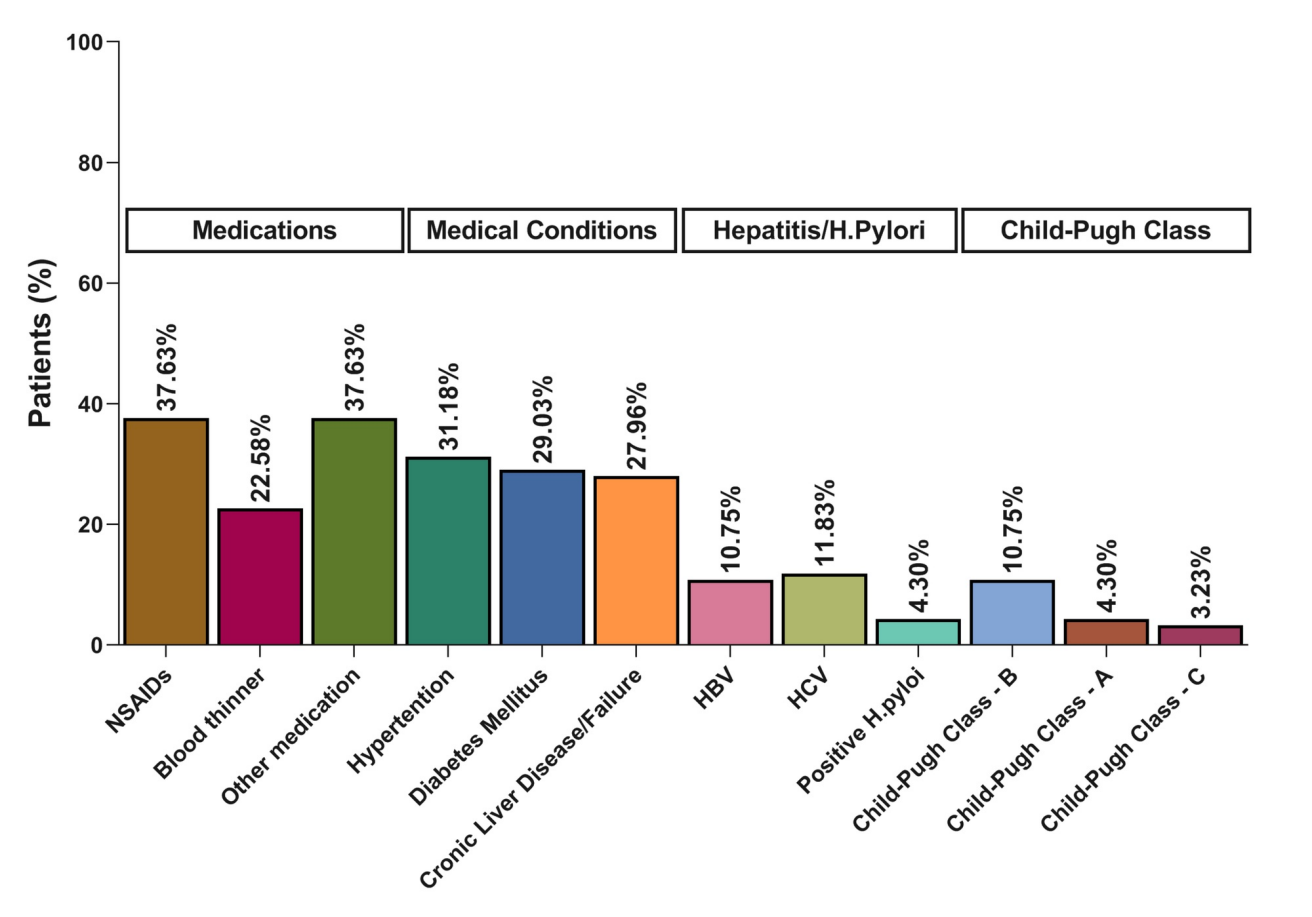
Risk factors for upper gastrointestinal bleeding among pilgrims NSAIDs: non-steroidal anti-inflammatory drugs; HBV: hepatitis B virus; HCV: hepatitis C virus

There were common risk factors observed among the included patients with UGIB. The most common factor was medications, especially NSAIDs (37.63%) and blood thinners (22.58%). Hypertension (31.18%), diabetes mellitus (DM) (29.03%), and chronic liver disease/failure (27.96%) were the most common chronic conditions in the studied population. Hepatitis B virus (HBV), hepatitis C virus (HCV), and *H. pylori* were all common conditions in the included patients (Figure 3).

## Discussion

Previously published literature shows that UGIB is the most common emergency medical admission related to gastroenterology, with an overall 28-day case-fatality in the range of 2-14%, and is associated with a significant burden on healthcare resources [9-11]. UGIB is commonly categorized as variceal (from esophageal or gastric varices) or non-variceal bleeding [12]. Non-variceal bleeding is more common and can be further subdivided by its causes [12].

In the current study, we aimed to define the most common causes and risk factors of UGIB. Our results showed that UGIB is more common in males and the elderly. The most common causes of UGIB were of a gastric origin, followed by causes of duodenal and esophageal origins. The most common risk factors were medications (NSAIDs and blood thinners), chronic medical conditions (hypertension, DM, and chronic liver disease/failure), hepatitis (HBV and HCV), and *H. pylori*.

These results are consistent with the previous literature. The incidence of UGIB is more among males compared to females, even at a young age, with a male-to-female ratio up to 6:1 in some studies [13,14]. It has also been shown that most of the patients are above the age of 40 years [13,15], which is similar to our results. Peptic ulcers have been reported as being the most common cause of UGIB, whether of gastric or duodenal origin, followed by esophageal varices or ulcers [16,17]. Medications like NSAIDs and blood thinners have been reported in many studies to be associated with a higher risk of UGIB, which is similar to our current findings [18,19]. This risk is mainly driven by enhancing mucosal damage and bleeding susceptibility, leading to a higher rate of UGIB and re-bleeding [18-22]. Furthermore, comorbidities have been found to increase the risk of UGIB; heart failure and diabetes were a risk factor in 5% and 4% of the UGIB patients, respectively. It was also found that patients with existing chronic conditions have a higher risk of UGIB compared to healthy individuals [23-25].

In Saudi Arabia, multiple studies have been conducted to identify morbidity and mortality rates among pilgrims. In the Islamic year of 1413 (1993), data of pilgrims with medical problems presenting to King Abdul Aziz, Madinah Al-Munawarah, were prospectively collected [26]. About 10.1% of the patients had gastrointestinal problems, and 16% of the mortalities were due to miscellaneous causes (including UGIB) [26]. In 2002, another prospective study of two locations in Al-Mashaer was conducted; it found that 1.9% of the patients among pilgrims were admitted with UGIB [27]. In 2003, a cross-sectional study of patients’ data from four hospitals in Mena and three hospitals in Arafat was done to review different causes of hospitalization [3]. The study found that gastrointestinal diseases were responsible for 4.2% of admissions and UGIB for 1.2% of hospitalizations [3]. In 2005, causes of hospitalization and mortality among pilgrims were prospectively collected; UGIB accounted for primary diagnosis in 34 patients, secondary diagnosis in five patients, and mortality in six patients.

The current study has some limitations. The number of included patients might be relatively too small to draw concrete conclusions. Another limitation is the retrospective nature of the data collected.

## Conclusions

The most common causes of UGIB are of a gastric origin, followed by those of duodenal and esophageal origins. The most common risk factors are male gender, old age, medications, and chronic medical conditions. A proper screening tool for patients at risk may help in the prevention of UGIB and decrease the burden of this health issue.
